# Cross Talk between H_2_O_2_ and Interacting Signal Molecules under Plant Stress Response

**DOI:** 10.3389/fpls.2016.00570

**Published:** 2016-04-28

**Authors:** Ina Saxena, Sandhya Srikanth, Zhong Chen

**Affiliations:** Natural Sciences and Science Education, National Institute of Education, Nanyang Technological UniversitySingapore, Singapore

**Keywords:** H_2_O_2_, ROS, abscisic acid, nitric oxide, biotic/abiotic stress, phytohormones

## Abstract

It is well established that oxidative stress is an important cause of cellular damage. During stress conditions, plants have evolved regulatory mechanisms to adapt to various environmental stresses. One of the consequences of stress is an increase in the cellular concentration of reactive oxygen species, which is subsequently converted to H_2_O_2_. H_2_O_2_ is continuously produced as the byproduct of oxidative plant aerobic metabolism. Organelles with a high oxidizing metabolic activity or with an intense rate of electron flow, such as chloroplasts, mitochondria, or peroxisomes are major sources of H_2_O_2_ production. H_2_O_2_ acts as a versatile molecule because of its dual role in cells. Under normal conditions, H_2_O_2_ immerges as an important factor during many biological processes. It has been established that it acts as a secondary messenger in signal transduction networks. In this review, we discuss potential roles of H_2_O_2_ and other signaling molecules during various stress responses.

## Introduction

In plants, reactive oxygen species (ROS) are continuously produced in different cellular compartments as byproducts of various metabolic pathways such as respiration and photosynthesis. All aerobic organisms use molecular oxygen as terminal oxidant during respiration. Oxygen is neither very reactive nor harmful, but it has the potential to be only partially reduced, leading to the formation of very reactive and therefore toxic intermediates like singlet oxygen (_1_O_2_), superoxide radical (${\text{O}}_{2}^{•-}$), hydrogen peroxide (H_2_O_2_) and hydroxyl radical (∙OH). All ROS are extremely reactive, causing damage to membranes and other cellular components. ROS also have strong affinities toward membrane, DNA, proteins, carbohydrates and lipids in plant cells (Anjum et al., 2015; Jajic et al., 2015). Hence, these molecules are constantly scavenged by different non-enzymatic and enzymatic detoxification mechanisms that are often confined to particular compartments (Alscher et al., 1997). It is important to remove ROS from the plant system in order to abate stress response, taking also into account that the final consequence of an eventual disequilibrium due to adverse environmental factors is the rapid increase of intracellular ROS levels, the so-called “oxidative burst" (Sharma et al., 2012). However, the balance between frequent production and scavenging of ROS may be disturbed by a number of adverse environmental factors such as light, temperature, drought, salinity, cold, heavy metal ions, UV exposure and water (Boyer, 1982). The usual external stress factors that affect ROS production in plants can be biotic (executed by other organisms) or abiotic (arising from changes in chemical of physical environment). However, in plants the constant regulation of the ROS concentration, including H_2_O_2_, is controlled by the enzymes such as catalase (CAT), ascorbate peroxidase (APX), glutathione peroxidase (GPX), glutathione *S*-transferases (GSTs), glutathione reductase (GR), and peroxyredoxin; and non-enzymatic compounds, like ascorbate, glutathione (GSH), α-tocopherol and flavonoids (Kapoor et al., 2015).

Recent studies have elucidated that under different stress conditions, plants react in a very complex manner which includes various physiological and cellular changes (Atkinson and Urwin, 2012). In order to combat stress response, plants use various signaling mechanisms derived from hormonal regulations. Nevertheless, recent studies indicate that plants also make use of ROS as signaling molecules for regulating development and various physiological responses. There is also increasing focus on ROS production and its integration with various hormonal signaling pathway in regulation of plant growth and stress tolerance (Xia et al., 2015).

Amongst all, superoxide and H_2_O_2_ are two ROS that have been given more importance in recent studies. The main focus of this review is on H_2_O_2_. H_2_O_2_ is freely diffusible across membranes, which enables it to diffuse the damage. It is relatively long lived and it acts as a central player in stress signal transduction pathways (Møller et al., 2007). Thenard was the first in 1818 to isolate H_2_O_2_ which later came across as a cell damaging molecule when accumulated at higher concentrations in the cell (Plaine, 1955). In early 90 s Ievinsh and Tiberg also predicted the role of H_2_O_2_ as a signaling molecule (Ievinsh and Tiberg, 1995). Based on earlier studies, it is certain that H_2_O_2_ is part of oxidative metabolism and is involved in various metabolism and signaling cascades which are essential for plant growth and development, such as development of root hair, reinforcement of plant cell wall, xylem differentiation, resistance enhancement, cell wall structural cross linking and cell wall loosening in stomatal control (Dempsey and Klessig, 1995).

H_2_O_2_ being a versatile molecule acts as an important signal at normal levels, whereas under abiotic or biotic stress conditions it induces oxidative stress. Its unique property of stability and less reactivity differentiates H_2_O_2_ from the rest of the ROS molecules (Yang and Poovaiah, 2002; Quan et al., 2008), thus making it a good signaling molecule. In plants, H_2_O_2_ works as a key factor in non-toxic level concentration. As a signaling molecule, it shows tolerance to biotic and abiotic stress by getting involved in various pathways (Mittler et al., 2004; Reczek and Chandel, 2015). At toxic concentration levels H_2_O_2_ showed involvement in programmed cell death (PCD; Dat et al., 2003). In a recent article, the dual nature of H_2_O_2_ has been studied where 600 μM H_2_O_2_ treatment caused an increase in the vase life of hybrid lily “Manissa,” while an increase in concentrations resulted in negative effects (Liao et al., 2012).

Several studies have indicated that H_2_O_2_ interplays with other signaling molecules such as abscisic acid (ABA) and ethylene which are important for plant development and senescence (Jubany-Mari et al., 2009; Chen et al., 2012). **Table 1** shows recent studies unveiling the mechanism by which H_2_O_2_ is involved in various biological processes. A recent study indicated the involvement of nitric oxide (NO) and H_2_O_2_ in salicylic acid (SA)-induced salvianolic acid B production in *Salvia miltiorrhiza* cell culture. Increase in H_2_O_2_ production has been observed by SA despite being inhibited by IMD (an inhibitor of NADPH oxidase) or DMTU (a quencher of H_2_O_2_) which further increases NO production and Sal B accumulation (Guo et al., 2014). In mung bean seedlings SA also plays roles in adventitious root formation and its effect on H_2_O_2_ accumulation has been observed. It has been concluded from the study that H_2_O_2_ accumulation acts as a downstream process in regulation with SA induced adventitious root formation (Yang et al., 2013).

**Table 1:** Recent studies showing the interrelation between H_2_O_2_ and its interaction with signaling molecules.

Hormone and interacting molecule	Plant	Interaction with H_2_O_2_	Reference
Nitric oxide	*Salvia miltiorrhiza*	Elicitation of SA for either H_2_O_2_ or NO was independent, and the elicited H_2_O_2_ or NO could act independently or synergistically to induce Sal B accumulation in SA-elicited cells.	Guo et al., 2014
Salicylic acid	Mung bean	Pretreatment of mung bean explants with N, *N*′-dimethylthiourea (DMTU), a scavenger for H_2_O_2_, resulted in a significant reduction of SA-induced ARF.	Yang et al., 2013
Ethylene	Tomato	Ethylene is a potentiator of the camptothecin-induced oxidative burst and subsequent PCD in tomato cells.	de Jong et al., 2002
Abscisic acid	Maize	H_2_O_2_ pretreatment may alleviate water loss and induce osmotic stress resistance by increasing the levels of soluble sugars, proline, and polyamines thus ABA and H_2_O_2_ production slightly decrease in maize seedlings under osmotic stress.	Terzi et al., 2014
Salicylic acid and nitric oxide	Wheat	SA and NO pretreatment reduces H_2_O_2_ level in wheat	Alavi et al., 2014
Jasmonate	Not applicable	Among the jasmonates, only 12-oxo phytodienoic acid (OPDA) suppressed H_2_O_2_-induced cytotoxicity. OPDA pretreatment also inhibited the H_2_O_2_-induced ROS increase and mitochondrial membrane potential decrease.	Taki-Nakano et al., 2014
Ethylene	*Arabidopsis*	H_2_O_2_ and ethylene interplay has an effect on AtERF73/HRE1 and ADH1 expression during the early stages of hypoxia signaling.	Yang, 2014
Abscisic acid and Calcium	*Arabidopsis*	ABA, H_2_O_2_, and Ca^2+^-induced stomatal closing were impaired in *Arabidopsis*.	Zou et al., 2015

H_2_O_2_ pretreatment in maize leaves significantly increased ABA content (Terzi et al., 2014). Pre-treatment of sodium nitroprusside (SNP) and SA in wheat seedlings decreased the effect of osmotic stress. It was also observed that pre-treatment of seedlings with methylene blue (a guanylatecyclase inhibitor) diminished the protective effects of both SA and SNP. Therefore, it is concluded that protective effect may only be limited to NO (Alavi et al., 2014). Another study on jasmonate revealed that 12-oxo phytodienoic acid is involved in reduced H_2_O_2_ accumulation (Taki-Nakano et al., 2014). Yang (2014), proposed a model describing three pathways in modulating the transcription factor *AtERF73/HRE1* which includes ethylene dependent, ethylene-independent/H_2_O_2_-dependent pathway, and an ethylene and H_2_O_2_-independent pathway. This study also proposes involvement of H_2_O_2_ and ethylene in *AtERF73/HRE1* and *ADH1* gene expression under stress. There is another study stating the involvement of ethylene in H_2_O_2_ accumulation during PCD (de Jong et al., 2002). In drought stress, calcium-dependent protein kinase (*CPK8*) has been involved in ABA-mediated stomatal regulation via *CAT3* (*CPK8*-interacting protein) activity. It also has been observed that *cpk8* and *cat3* plants showed reduced CAT activity and higher H_2_O_2_ accumulation (Zou et al., 2015).

Guo et al. (2014) studied the possibility of SA involvement in H_2_O_2_ and NO induced salvianolic acid B accumulation; where the main function of NO is to downstream SA signaling which results in reduced oxidative stress (Alavi et al., 2014). Exogenous application of H_2_O_2_ and its induction in high light showed different effects on gene expression (Olemiec et al., 2014). It was shown that H_2_O_2_ could be involved in the signaling of plant growth regulators such as ethephon (Chen et al., 2012). The application of ethephon results in an elevation in H_2_O_2_ levels, which is accompanied by the increased expression of sweet potato CAT.

## H_2_O_2_ Production In Plant Cells And Its Reactivity In Different Cellular Compartments

**FIGURE 1:**
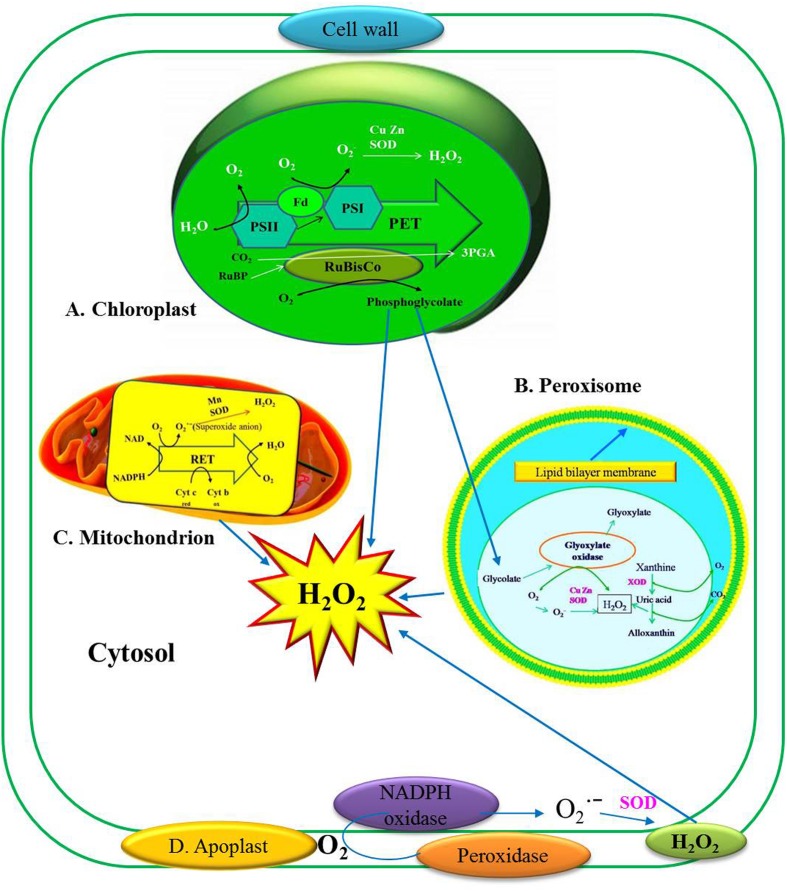
**Production of hydrogen peroxide (H_2_O_2_) in plant cells and their reactivity in various cellular components. (A)** H_2_O_2_ production in Chloroplast at the site of PSI and PSII. **(B)** H_2_O_2_ production in Peroxisomes. XOD (Xanthine oxidase), GOX (glycolate oxidase). **(C)** H_2_O_2_ production in Mitochondria. **(D)** H_2_O_2_ production in Apoplast (Figure modified from Jajic et al., 2015).

H_2_O_2_ is produced in photosynthesizing cells, majorly in peroxisomes (photosynthetic carbon oxidation cycle) and minorly in choroloplast, mitochondria (respiratory electron transport chain) (**Figure 1**), the endoplasmic reticulum (ER), nucleus and plasma membranes (del Río et al., 2006; Bhattacharjee, 2012). Peroxisomes and chloroplasts may accumulate 30–100 times higher H_2_O_2_ as compared to mitochondria (Hossain et al., 2015). H_2_O_2_ production occurs during the oxidative burst when reduction of molecular oxygen (O_2_) into superoxide anion (${\text{O}}_{2}^{•-}$) occurs (Sutherland, 1991). At the cell wall this ${\text{O}}_{2}^{•-}$ undergoes spontaneous dismutation at a higher rate and at an acidic pH. Nicotinamide adenine dinucleotide (NADH) undergoes oxidation to form NAD^+^ by cell wall peroxidase with further reduction of O_2_ to ${\text{O}}_{2}^{•-}$ resulting in the production of O_2_ and H_2_O_2_ (Bhattacharjee, 2005). In apoplast, amine oxidase and germin-like oxidase have been proposed to generate H_2_O_2_ (Bolwell and Wojtaszek, 1997). Cell membrane NADPH-dependent oxidase serves as a H_2_O_2_ source. H_2_O_2_ production occurs in the cell via reaction between the oxygen molecules (O_2_), forming superoxide anion (${\text{O}}_{2}^{•-}$). During the stress response O_2_ is reduced to ${\text{O}}_{2}^{•-}$ which undergoes spontaneous dismutation (Sutherland, 1991). ${\text{O}}_{2}^{•-}$ is also catalyzed and reduced by superoxide dismutase (SOD) and protein kinase C (PKC) to form H_2_O_2_ (Scandalios, 1993). SOD enzyme catalyzes ${\text{O}}_{2}^{•-}$ which mainly occurs in cytosol, chloroplast and mitochondria (Scandalios, 1993). Rather than superoxide disproportionation, H_2_O_2_ is also produced by ${\text{O}}_{2}^{•-}$ reduction by reductants such as ferredoxins, thiols, ascorbate (Asada and Takahashi, 1987). PKC also shows involvement in H_2_O_2_ production. OH is produced in the reaction of H_2_O_2_ with Fe^2+^ (Arora et al., 2002). To maintain the balance between H_2_O_2_-scavenging enzymes and SODs, equilibrium for H_2_O_2_ concentration in cells should be attained (Mittler et al., 2004).

H_2_O_2_ production in chloroplasts originates from several locations and occurs mainly in Chl associated with the photosynthetic electron transport chain (PET) which is the primary source of O_2_. In chloroplast, molecular oxygen (O_2_) uptake is associated with photoreduction of O_2_ to superoxide radical (${\text{O}}_{2}^{•-}$). Singlet oxygen (_1_O_2_) is produced by energy transfer to triplet oxygen (_3_O_2_) in photosystem II (PSII). Photosystem II excitation results in the oxidation of water (H_2_O) to O_2_ (**Figure 1A**). The reductant formed by this process donates electrons (e^–^) to plastoquinone (PQ). These e^–^ eventually passes through the cytochrome *f* (Cyt *f*) complex, plastocyanin (PC), and then to photosystem I (PSI). The PET chain includes a number of enzymes on the reducing (acceptor) side of photo system I (PSI): Fe–S centers, reduced thioredoxin (TRX), and ferredoxin. When ferredoxin (Fd) is over reduced during photosynthesis electron transfer, electron may be transferred from photosystem I (PSI) to O_2_ to form ${\text{O}}_{2}^{•-}$ by the process called Mehler reaction. The ${\text{O}}_{2}^{•-}$ formed then undergoes dismutation to hydrogen peroxide (H_2_O_2_) either spontaneously or facilitated by SOD (**Figure 1A**).

In plants, the main function of peroxisome is photorespiration which involves O_2_ uptake (light-mediated) and the emission of CO_2_ in simulation with H_2_O_2_ formation (Dat et al., 2000). H_2_O_2_ production in peroxisome results from the oxygenase activity of ribulose-1,5-bisphosphate carboxylase/oxygenase (RuBisCO) (Noctor et al., 1999). It is being stated that plants exposed to favorable conditions for oxygenation are more subjected to produce H_2_O_2_ (Foyer and Noctor, 2000). H_2_O_2_ is generated during the oxidation of glycolate in the C2 cycle of peroxisomes (**Figure 1B**). Production of H_2_O_2_ is attributed to the matrix-localized enzyme, xanthine oxidase (XOD), which catalyses the oxidation of xanthine or hypoxanthine to uric acid, and is a well-known producer of ${\text{O}}_{2}^{•-}$ in the process. This ${\text{O}}_{2}^{•-}$ is then converted into H_2_O_2_ by Cu, Zn-SOD (**Figure 1B**) (Corpas et al., 2001). An NAD(P)H-dependent ${\text{O}}_{2}^{•-}$ production site which uses ${\text{O}}_{2}^{•-}$ as an electron acceptor is present in the peroxisomal membrane and it releases the ${\text{O}}_{2}^{•-}$ into the cytosol. This site appears to be formed by a small ETC which is composed of a flavoprotein NADH and Cyt *b* (del Río et al., 2006).

Two major mitochondrial sites for superoxide radical production in electron transport chain are cytochrome bc1 complex and NAD(P)H dehydrogenases (Moller, 2001). The mitochondrial respiratory electron transport (RET) harbors electrons with sufficient free energy to directly reduce O_2,_ which is considered as a primary source of H_2_O_2_ generation (**Figure 1C**). This direct reduction of O_2_ to ${\text{O}}_{2}^{•-}$ takes place in the flavoprotein region of the NADH dehydrogenase segment, which is responsible for the O_2_ consumption. During RET, the oxygen radical is markedly enhanced in the presence of antimycin A, which blocks the electron flow after ubiquinone (UQ). Autooxidation of reduced UQ results in the production of ${\text{O}}_{2}^{•-}$ which is a major precursor of H_2_O_2_ production in UQ location of the RET. Completely reduced UQ donates e^–^ to cytochrome c (Cyt *c*) and leaves an unstable, highly reducing semiquinone species, which would normally reduce cytochrome *b* (Cyt *b*), which instead reduces the O_2_ to ${\text{O}}_{2}^{•-}$ (**Figure 1C**). This ${\text{O}}_{2}^{•-}$ is further reduced by manganese SOD (Mn-SOD) dismutation to H_2_O_2_ (Moller, 2001).

Some other sources of H_2_O_2_ production are plasma membrane, cytoplasm and the extracellular matrix (ECM). There are various enzymes responsible for H_2_O_2_ production in plasma membrane (Vianello and Macri, 1991). H_2_O_2_ production in cytoplasm occurs by the electron transport chain which is associated with the ER. This oxidation and hydroxylation process involve cytochrome P450 and cytochrome P450 reductase whereas, fatty acid desaturation involves cytochrome b_5_ and cytochrome b_5_ reductase, which donate electrons for H_2_O_2_ formation (Bartosz, 1997; Mittler et al., 2004).

Studies have shown that NADPH oxidase at the plasma membrane in the plant cell is involved in plant defense reactions to pathogen attack (Torres et al., 2002) and in response to various abiotic stresses (Kwak et al., 2003). The NADPH-dependent oxidase system sometimes referred to as *rboh* (for respiratory burst oxidase homolog), similar to that present in mammalian neutrophils, has received the most attention. It catalyzes the production of ${\text{O}}_{2}^{•-}$ by one-electron reduction of oxygen using NADPH as the electron donor (Desikan et al., 2003; Mahalingam and Federoff, 2003; Apel and Hirt, 2004). The superoxide anion radical is most likely located in the apoplastic space and is converted to H_2_O_2_ either spontaneously or by extracellular SOD (Karpinska et al., 2001; Bolwell et al., 2002) (**Figure 1D**). There are other plant species which NADPH oxidase or *rboh* genes have been cloned (Desikan et al., 2003).

## Abscisic Acid And Interaction With H_2_O_2_

Abscisic acid is one of the crucial phytohormones which play important roles under various environmental cues. It accumulates as a response to stressful environmental conditions such as dehydration, cold temperature or shortened day length. The application of ABA plays fundamental role in different physiological processes and biological pathways such as seed dormancy and delay in germination, development of seeds, promotion of stomatal closure, embryo morphogenesis, synthesis of storage proteins and lipids, leaf senescence and also defense against pathogen (Swamy and Smith, 1999).

**FIGURE 2:**
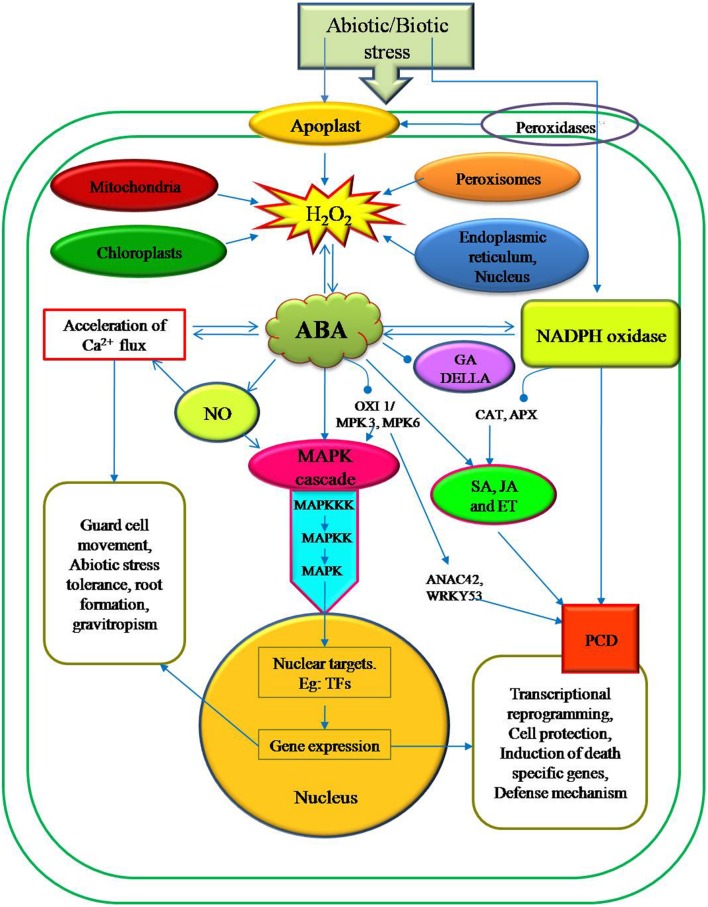
**Schematic representation of H_2_O_2_ generation in various intra-and extra cellular sites and its involvement in the various signaling pathways associated with the regulation of defense gene expression in plant cells.** ABA is extensively associated with wide range of abiotic stress signals and administers with growth and development processes in plants. In contrast to ABA, other phytohormones such as SA, GA, JA, and ethylene have significant role during biotic stress tolerance in plants. Nevertheless, ABA is an important signaling molecule in H_2_O_2_ production and activating the MAPK cascade by interacting with other plant hormones.

It has been reported that the ABA signaling pathway is identified as a central regulator of abiotic stress response in plants, triggering major changes in gene expression and adaptive physiological responses (**Figure 2**). Recently, MAPK (mitogen activated protein kinase) cascades have also been shown to be implicated in ABA signaling. External ABA treated plants induced the transcriptional regulations, protein accumulation and stability, and kinase activity of several components of distinct MAPK signaling cascades in many plant species. These existing evidences suggest that MAPK cascades are actively involved in several ABA responses, including abiotic stress defense mechanisms and guard cell signaling (Zhang et al., 2007; Jammes et al., 2009; Zong et al., 2009). Rodriguez et al. (2010) reported that the MAPK cascade is activated by the exogenous H_2_O_2_ which in turn is mediated by the hormones like ABA, Jasmonic acid (JA) and SA.

In plants, MAPK cascades are generally organized pathways in which the upstream activated MAPK kinase kinase (MAPKKK) undergoes the sequential phosphorylation and subsequent activation of downstream MAPK kinases (MAPKKs) and MAPKs. This MAPK cascades translate incoming environmental signals into post-translational modifications of target proteins that ultimately reorganize gene expression and stress adaptation. In *Arabidopsis*, H_2_O_2_ activates the MAPKs (MPK3 and MPK6) via the MAPK kinase kinase (MAPKKK) ANP1 (Kovtun et al., 2000). MKP2 is a key regulator of the MPK3 and MPK6 networks that are involved in controlling both abiotic and biotic stress responses (Zhou et al. (2012). Treatment with ABA in *Arabidopsis* plants induced the transcriptional regulation of MPK3, MPK5, MPK7, MPK18, MPK20, MKK9, MAPKKK1 (ANP1), MAPKKK10 (MEKK3), MAPKKK14, MAPKKK15, MAPKKK16, MAPKKK17, MAPKKK18, MAPKKK19, *Raf6*, *Raf12*, and *Raf35* (Menges et al., 2008; Wang et al., 2011) signifying a promising role in ABA signaling. Recently, Jammes et al. (2009) used a cell type-specific functional genomics approach and identified that the MAPKs, MPK9 and MPK12 were preferentially expressed in guard cells. It was also reported that MPK12 were activated by ABA and H_2_O_2_. MPK9 and MPK12 mediate ABA signals in guard cells (Montillet et al., 2013). In *Arabidopsis*, a T-DNA *oxi1* null mutant is impaired in the activation of the two MAPKs, MPK3 and MPK6, upon oxidative stress (Rentel et al., 2004), suggesting that serine/threonine kinase OXI1 functions downstream of ROS but upstream of the MAPK module. H_2_O_2_ also increases the expression of the *Arabidopsis* nucleoside diphosphate (NDP) kinase 2 (Moon et al., 2003). H_2_O_2_ accumulation was reduced by the over expression of At-NDPK2 and enhanced tolerance to multiple stresses, including cold, salt, and oxidative stress. Moreover, the MAPKs, MPK3 and MPK6 are activated by H_2_O_2_ induced NDPK2. Gechev et al. (2005) identified a transcription factor ANAC42 and reported that it was highly regulated by H_2_O_2_ which in turn is achieved through OXI1/MPK3 and MPK6 activation (**Figure 2**). Interestingly, ANAC42 plays a role in senescence and H_2_O_2_ induced PCD (Fujii, 2014). Also, the MAPKKK can interact directly with WRKY53, a transcription factor that involved in senescence induced PCD (**Figure 2**), thus bypassing the downstream kinases (Miao et al., 2006). Nevertheless, it is clear from the past reports that MPK3 and MPK6 are two important integrating points of signals from cellular developmental programs and the external environment changes (Wang et al., 2008). Even though most of the connections between ABA and MAPKs are poorly understood, it is evident that these pathways are part of the complex cellular signaling network of plants to integrate various environmental signals (Danquah et al., 2014).

One of the most important functions of ABA is to induce stomatal closure by reducing the turgor of guard cells under water deficit. There is evidence that H_2_O_2_ may function as an intermediate in ABA signaling in *Vicia faba* guard cells (Pei et al., 2000). There are reports which suggest that H_2_O_2_ is one of the major ROS and plays an important role as a second messenger in ABA induced stomatal closure (Pei et al., 2000; Miao et al., 2006). While ABA induced the synthesis of H_2_O_2_ and superoxide under stressful condition and caused oxidative stress (Guan et al., 2000). However, ABA is a natural growth regulator which accumulates in plants under plant stress conditions (Zheng et al., 2010). Results of (Zhang et al., 2001) provide evidence for H_2_O_2_ involvement in ABA induced stomatal movement in *V. faba.* Major findings of this study showed stomatal closure by exogenous application of H_2_O_2_ with ABA whereas, reverse action of H_2_O_2_ generation by scavenging its activity results in reduced stomatal closure (Guan et al., 2000). Overproduction of ABA induces H_2_O_2_ accumulation which enhances the stomatal closure by activating plasma membrane calcium channels (Pei et al., 2000). Plasma membrane-located NADPH oxidases Atrboh D and Atrboh F were found to be required for ABA-induced stomatal ROS production (Kwak et al., 2003). It has been reported that during oxidative burst, NADPH oxidase can trigger Ca^2+^, and mitogen-activated protein kinase (MAPK) signaling pathways as well as suppress the hormone signal transfer routes such as SA, JA, and ethylene (Overmyer et al., 2003; Evans et al., 2005) (**Figure 2**). Abiotic stress condition enhances ABA/gibberellic acid (GA) ratio supporting DELLA protein accumulation which in turn lowers H_2_O_2_ levels (Considine and Foyer, 2014). In rice seeds, ABA decreased ROS production, which leads to a repression of ascorbate and GA accumulation (Ye et al., 2012). It was also reported that H_2_O_2_ is involved in GA/ABA signaling in barley aleurone cells (Ishibashi et al., 2012). Calcium and calmodulin (CaM)-dependent protein kinase (CCaMK) belongs to calcium/CaM-dependent protein kinase superfamily (Harmon et al., 2000); activation by free Ca^2+^ and Ca^2+^ bound to CaM has been indicated to be involved in ABA signaling during abiotic stress responses (Yang et al., 2011) and ABA-induced antioxidant defense by functioning upstream of ABA-activated MAPK (Shi et al., 2014).

These available pieces of evidence clearly indicate that the ABA is a key hormone in inducing abiotic stress responses. Downstream events mediated by MAPK cascade, alterations in Ca^2+^ fluxes and the activation of ion channels changes the redox state of the cell. All these actions lead to transcriptional reprogramming, which results in target gene expression such as cell death specific nucleases and proteases, and eventually PCD (**Figure 2**). Most of the genes (NADPH oxidases, SOD and extracellular peroxidases) expressed in the early signals are involved in the ROS generation, essential for triggering PCD. While other genes are responsible for maintaining ROS levels (CAT and APXs) (Gadjev et al., 2008) (**Figure 2**).

## Calcium And Interaction With H_2_O_2_

Calcium (Ca^2+^) is important for robust cell wall formation. It is also crucial for the root system and in young, growing cells. Alteration in Ca^2+^ level is observed under shifting environmental conditions, including changes in light, temperature, and drought (Mahajan and Tuteja, 2005). Ca^2+^ is significant for cross-linking acidic pectin residues and permeability of the plasma membrane. As a secondary messenger, Ca^2+^ concentration is balanced by storing it in vacuoles and is released whenever necessary (Mahajan and Tuteja, 2005). It is indispensable for all important signaling pathways. Plant signaling network has the capability to alter its functioning under various environmental challenges. The plant cell primary response under stress condition is a modification in plasma membrane permeability leading to calcium and proton influx that appears to be necessary and sufficient for the induction of H_2_O_2_ (Pei et al., 2000). Plant metabolism involves various Ca^2+^/CaM proteins having different functions out of which some are involved in H_2_O_2_ signaling such as NAD kinase (Harding et al., 1997). CAT is also important for H_2_O_2_ regulation and its deficiency can lead to H_2_O_2_ accumulation (Gechev et al., 2004). H_2_O_2_ and Ca^2+^ interrelation study has been shown by Yang and Poovaiah (2002) in *Arabidopsis*. Another study on *Arabidopsis* seedlings suggests the role of H_2_O_2_ in biphasic Ca^2+^ elevation, with the first peak located in cotyledons and the second in the root (Rentel and Knight, 2004). The antioxidant system may also be considered as a target of Ca^2+^ influence, for example, the efficiency of H_2_O_2_ scavenging in *Arabidopsis* plants depends on the peroxisomal Ca^2+^ concentration (Costa et al., 2010).

Continuous Ca^2+^ invasion is most importantly required for H_2_O_2_ accumulation which also activates NADPH oxidase located in the plasma membrane (Lamb and Dixon, 1997). There is another study suggesting the role of biphasic (Ca^2+^) and H_2_O_2_ in aequorin tobacco cell culture’s expression (Lecourieux et al., 2002). Pollen tube growth has been enhanced by H_2_O_2_ regulated spermidine oxidase, which also induces Ca^2+^ channel (Wu et al., 2010). H_2_O_2_ involvements in Ca^2+^ influx via Ca^2+^ permeable channel and partial stomatal closure were observed in the study (Pei et al., 2000). Significant induction in nuclear and cytosolic Ca^2+^ level by free sphingoid Long Chain Base (LCB) sphinganine has been observed in simulation with decreased accumulation of H_2_O_2_ in tobacco cells (Lachaud et al., 2011). Later studies have revealed that CAT can scavenge H_2_O_2_ production which is likely mediated by Ca^2+^ homeostasis in *Arabidopsis* (Suzuki et al., 2011). In this case, cytoplasmic Ca^2+^ was shown to bind to *rboh* at the N-terminal region and thus to promote the activation of *rboh* and produce H_2_O_2_ (Takeda et al., 2008). H_2_O_2_ mediated rapid gene expression (*LeCDPK1*) in tomato leaves has been observed (Chico et al., 2002) whereas, H_2_O_2_ treatment in wheat plant also leads to enhanced responsiveness in eight out of 20 studied calcium-dependent protein kinase (CDPKs) (Li et al., 2008). Induction in gene expression (*GST1*) by Ca^2+^response in association with H_2_O_2_ may be due to changes in redox balance (Rentel and Knight, 2004).

## Nitric Oxide And Interaction With H_2_O_2_

Increasing evidence based on experiments has shown a vital role of NO in protecting plants against stress conditions (Wink et al., 1993). It is generated in higher plants through oxidative (arginine or hydroxylamine-dependent) and reductive (nitrate-dependent) pathways (Gupta et al., 2011). NO being part of various physiological processes in plant system makes it one of the major signaling molecules. Initially NO was considered to be a toxic gas. However, this idea changed after the discovery of the signaling role of NO in regulating the cardiovascular system (Skovgaarda et al., 2005). One of the major areas in the study of NO is its involvement in coordinating several defense responses during both biotic and abiotic stress conditions in the plants. In the past 2 decades, much focus was given to NO related studies toward its role in plant defense system under oxidative stress. Studies on adaptive mechanisms of plants have shown an increased basal level of NO in water and heat stressed plants, suggesting its importance in abating stress (Leshem and Haramaty, 1996; Leshem et al., 1998). The defensive mechanism of NO in plants under oxidative stress is associated with its ability to function as an antioxidant by directly scavenging the ROS, thus reducing cellular damage (Romero-puertas and Sandalio, 2016) and acting as a signaling molecule which eventually results in changes in gene expression (Lamattina et al., 2003).

A study focused on H_2_O_2_ generation in simultaneous correlation with NO production was shown during the hypersensitive response (HR) in which both cooperates to trigger hypersensitive cell death (Delledonne et al., 2001). The function of NO is tightly linked to ROS and it has a half-life of only a few seconds, once produced, interacts rapidly with ROS, giving rise to a number of reactive nitrogen species, such as nitrogen dioxide (NO_2_), which degrades to nitrite and nitrate in aqueous solutions (Neill et al., 2008; Bellin et al., 2013). There were studies showing involvement of both NO and H_2_O_2_ in bacterially induced PCD in soybean where both signals work synergistically (Delledonne et al., 1998) and in *Arabidopsis* they work as additive (Clarke et al., 2000). H_2_O_2_ formation may occur via superoxide radical (${\text{O}}_{2}^{•-}$). There is a probability that NO reacts with ${\text{O}}_{2}^{•-}$ to form highly reactive peroxynitrite anion ONOO- and subsequent cellular effects may then be induced by peroxynitrite (Bellin et al., 2013).

In mammals, NO has been shown to react with glutathione to form *S*-nitrosoglutathione (GSNO) which serves as a systemic source of NO and a similar situation has been suggested for plants (Durner and Klessig, 1999). It is clear that both H_2_O_2_ and NO can mediate transcription, but the involved processes remain unclear. There is a possibility of both H_2_O_2_ and NO having a direct effect on transcription factors by *S*-nitrosylation and oxidation of cysteine. A recent study suggests characterization of redox-sensitive factor in yeast where H_2_O_2_ oxidation alters the activity of this protein (Delaunay et al., 2000). Phosphorylation of cascade such as the mitogen-activated protein kinases (MAPK) is suggested to play another important role on H_2_O_2_ and NO. There is another study on tobacco Bright Yellow-2 (TBY-2) cells, suggesting an involvement of both H_2_O_2_ and NO in the activation of PCD, and treatment of scavenger for both the signaling molecules restores the cell viability (De Pinto et al., 2006).

In a new study, the cloning of rice *NOE1*, a gene whose knockout enhances NO production, revealed that this is indeed the rice CAT OsCATC (Lin et al., 2011). Increase in leaf H_2_O_2_ content leads to a characterization of mutant *NOE1* which in turn leads to nitrate reductase (NR) dependent NO production. Increased H_2_O_2_ concentrations provoked by ABA may in turn trigger NO generation by NR and nitrogen oxide synthase (NOS)-like enzymes (Neill et al., 2008). NO accumulation under abiotic stress is similar to the events seen in H_2_O_2_ production (Wang et al., 2014). In *Arabidopsis*, both H_2_O_2_ and NO showed similar function which influences the induction and reduction in root growth stimulated by various concentrations of nucleotides (Clark et al., 2010). He et al. (2013) reported that NO and H_2_O_2_ are also involved in the stimulation of stomatal closure in *Arabidopsis* in response to ultraviolet-B exposure. Exclusion of H_2_O_2_ with antioxidants or inhibition of its synthesis by inhibiting NADPH oxidase activity prevents NO generation and stomatal closure. Wang et al. indicated the idea that H_2_O_2_-induced synthesis of NO might be mediated by MPK6 in *Arabidopsis* (Wang et al., 2010).

## Salicylic Acid And Interaction With H_2_O_2_

Salicylic acid is one of the key phytohormones involved in both abiotic (Kunihiro et al., 2011; Drzewiecka et al., 2012; Liu et al., 2012) and biotic (Vlot et al., 2008; Dempsey et al., 2011) stress adaptation. The discovery of the salicylate role in thermotolerance during potato tissue culture research was mere coincidence. Inclusion of the artificial SA analog acetyl salicylic acid (ASA) in the culture medium of microplants of potato (*Solanum tuberosum* L.) causes potentially useful effects such as tuberization. It has been shown to play a central role as a signaling molecule involved in both local defense reactions and in the induction of systemic resistance (Durner and Klessig, 1999; Herrera-Vásquez et al., 2015). Another important aspect is gene regulation of antifungal hydrolases by SA, such as pathogenesis-related (PR) encoding PR1 and PR proteins which target to the plant cell wall (Durner and Klessig, 1999). Reduced synthesis of SA in transgenic plants due to disruption of SA pathways results in vulnerability toward fungal (*Phytophtora parasitica, Cercospora nicotianae*), bacterial (*Pseudomonas syringae*), and viral (*tobacco mosaic virus*) pathogens (Delaney et al., 1994).

There are various reports suggesting involvement of SA in various biotic and abiotic stresses (Wan et al., 2012; Miura and Tada, 2014; Herrera-Vásquez et al., 2015). Various environmental factors such as temperature, salinity, drought and high light exposure are responsible for ROS generation in cell organelles (peroxisomes, chloroplast) (Apel and Hirt, 2004; Holuigue et al., 2007). SA can be directly or indirectly involved in signaling pathways and interplays with ROS and GSH in stressed plants (Mateo et al., 2006; Lee and Park, 2010; Herrera-Vásquez et al., 2015). Under drought stress, increased level of SA has been observed in oat plants (Sánchez-Martín et al., 2015), whereas another study also stated the same condition in peroxisome and chloroplast for the gene *cat2* knockout (Chaouch et al., 2010) and thylakoidal ascorbate PRX gene silencing (Maruta et al., 2012; Noshi et al., 2012), respectively. H_2_O_2_ stimulated PCD, SA accumulation and sesquiterpene production in cultured cell suspensions of *Aquilaria sinensis* (Liu et al., 2014). H_2_O_2_ production induced SA up-regulated the mRNA transcription of heat shock protein (Hsp) genes through *AtHsfA2*, a key component of acquiring thermotolerance in *Arabidopsis* (Nie et al., 2015).

Salicylic acid and H_2_O_2_ interrelation is suggested by Leon, since the pathway involved benzoic acid (the immediate precursor of SA) leads to activation of benzoic acid 2-hydroxylase which is H_2_O_2_ dependent (León et al., 1995). Relative pathway of this case suggest that H_2_O_2_ production in cell organelles (peroxisome and chloroplast) induces SA synthesis, and leads to protective mechanism such as stomatal closure and cell death. Salicylate can increase H_2_O_2_ levels in plant tissues (Rao et al., 1997; Dat et al., 1998), on the contrary SA accumulation can be induced by elevated H_2_O_2_ levels (Chamnongpol et al., 1998). The germination of *sid2* seeds under high salinity is hypersensitive to H_2_O_2_, but the physiological concentrations of SA modulate antioxidant activity to prevent oxidative damage (Lee et al., 2010). There is another study suggesting exogenous application of SA relieves Cd toxicity by reducing the H_2_O_2_ accumulation in root apoplasts of the legumes *Phaseolus aureus* and *Vicia sativa* (Zhang et al., 2011).

The HR to pathogens exhibits an early ‘oxidative burst’ of superoxide which rapidly dismutates to H_2_O_2_. This mechanism involves key interactive roles for SA and H_2_O_2_, as the HR was impaired in tobacco plants with an H_2_O_2_-inducible SA-hydroxylase transgene (Mur et al., 1997). It is considered that mammalian plasma membrane NADPH oxidase is a homolog of oxidative stress enzyme (Keller et al., 1998) and it may be that this enzyme is potentiated by SA (Kauss and Jeblick, 1996). Increased accumulation of SA and enhancement in H_2_O_2_ concentration in simultaneous pathogenesis gene induction were observed in *GR1* dependent glutathione (Mhamdi et al., 2010).

## Ascorbic Acid And Interaction With H_2_O_2_

Ascorbic acid (AsA) is a critical water soluble phytohormone found in plant and animals (Levine, 1986; Sies and Stahl, 1995). It acts as a signal for plant growth and development, and regulates cell division, growth and signal transduction (Kerk and Feldman, 1995; Smirnoff and Wheeler, 2000). In the mitochondria plants synthesize AsA which is then transported to other parts of the plants (Shao et al., 2008). There can be a direct or indirect reaction of H_2_O_2_ with AsA, which is catalyzed by APX. APX is responsible for scavenging H_2_O_2_ hyperaccumulation found in higher plants (cytosol, chloroplast and mitochondria) (Mittler and Zilinskas, 1991).

**FIGURE 3:**
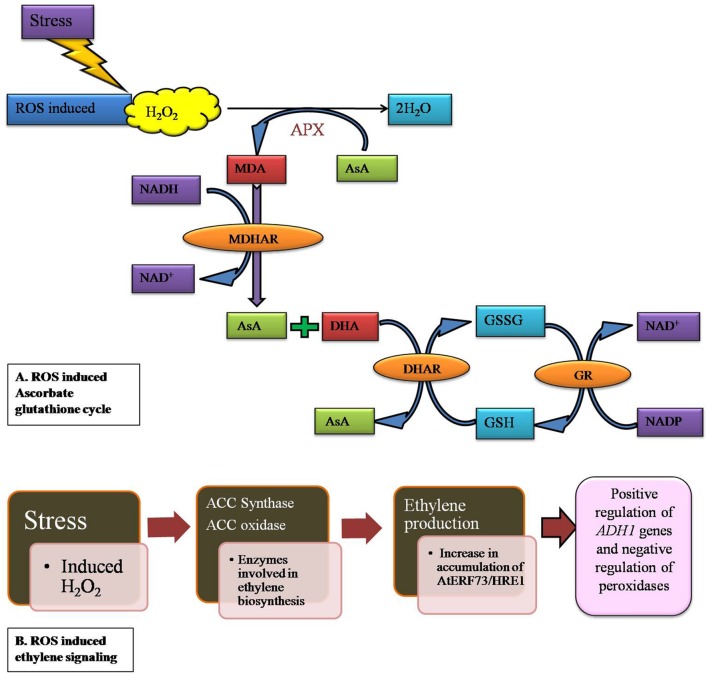
**H_2_O_2_ induced antioxidant mechanism in plants which involved in detoxification process. (A)** Plants synthesize ascorbic acid in the mitochondria and transported to other parts of the plants. Ascorbate peroxidase (APX) uses AsA as a substrate to reduce H_2_O_2_ to H_2_O in the ascorbate-glutathione cycle and generate mono dehydroascorbate (MHA), which further dissociate to AsA and dehydroascorbate (DHA). **(B)** Ethylene responsiveness in gene induction upon H_2_O_2_ accumulation in plant cells. Enzymes which involved in ethylene production activate an ethylene responsive group (AtERF73/HRE1). These genes are involved in negative regulation of peroxidases which in turn reduces H_2_O_2_ accumulation_._

H_2_O_2_ detoxification can be done by various antioxidants in peroxisomes such as CAT in the matrix, APX and monodehydroascorbate reductase (MDAR) in association with AsA, resulting in a decrease in the accumulation of H_2_O_2_ (Yamaguchi et al., 1995; Karyotou and Donaldson, 2005). In the chloroplast stroma, where the pH is higher during the day time, there is a consequence of AsA consumption during H_2_O_2_ reduction. A rate limiting amount of dehydroascorbate reductase (DHAR) efficiently catalyzes the recycling of AsA. The signaling function of H_2_O_2_ in guard cells is controlled by the rate of its production and the rate of its removal, in which AsA and DHAR play a critical role. The slower responsiveness of guard cells of DHAR over expressing tobacco allows more ozone to diffuse into the leaf interior (Chen and Gallie, 2004). However, the increase of AsA content in all cells and consequent increase in their ability to detoxify entered ozone, reduce the oxidative load of the leaf (i.e., lower levels of foliar and apoplectic H_2_O_2_). From past reports, it is clear that the oxidative stress induced ROS level increases monodehydroascorbate (MDA) accumulation which is being converted into L-ascorbate (AsA) and dehydroascorbate (DHA) (**Figure 3A**). The accumulated H_2_O_2_ is reduced to H_2_O by oxidation of AsA to MDA radical, which is catalyzed by APX. The MDHA is subsequently reduced back to AsA by either ferredoxin reduction or NAD(P)H catalyzed monodehydroascorbate reductase (MDHAR) (Sano et al., 2005). In GPX cycle, similar to APX, GPX uses GSH as a reducing agent to detoxify H_2_O_2_ to H_2_O. In addition to GPX, the organellar redox state is regulated by different enzymatic antioxidants like GR, MDHAR in addition to GPX. Disproportionation in L-ascorbate and MDHA is maintained by GSH (Venkatesh and Park, 2014) (**Figure 3A**).

## Jasmonate And Interaction With H_2_O_2_

Phytohormones act as a major factor responsible for plant growth and development. Oxylipin is considered to be one of the most important signaling molecules, i.e., plant hormone JA. Due to the unique physiological properties and abundance, jasmonate and its derivate (methyl jasmonate) came into the limelight as bioactive in nature. Chloroplast membrane is considered to be the initial site for JA synthesis where membrane phospholipids acts as a source for alfa-linolenic acid (C18:3) and hexadecatrienoic acid (C16:3) production (Ishiguro et al., 2001). Major pathway for JA synthesis in plants is supposed to be an octadecanoid pathway with the involvement of alfa-linolenic acid as a substrate (Mueller et al., 1993).

The defensive mechanism of JA has been observed in tomato against tobacco hornworm larvae (Howe et al., 1996) whereas in *Arabidopsis* against the fly *Bradysia* (McConn et al., 1997). There are studies confirming the role of JA as protective agent against pathogen (*Pythium mastophorum)* attack in *Arabidopsis* (Vijayan et al., 1998). Hu et al. (2003), came across with the result that both H_2_O_2_ and JA are primary signaling molecules during the cellular response involved in saponin biosynthesis mediated by oligogalacturonic acid (OGA) which also leads to the H_2_O_2_ mediated upregulation of JA. JA derivate (methyl jasmonate) has also been studied for its involvement in the induction of H_2_O_2_ accumulation in parsley suspension-cultured cells (Kauss et al., 1994), whereas another study suggested its role in inducing defensive genes of tomato (Orozco-Cardenas et al., 2001).

Jasmonic acid induces glutathione, an important antioxidant for redox balance. Increased expression of nuclear factor erythroid 2-related factor 2 (*NrF2*) has also been observed, which reduces the ROS level induced by H_2_O_2_ (Taki-Nakano et al., 2014). In association with this study, increased expression of glutamyl cysteine ligase with an increase in *NrF2* helps in regulating enzymes reducing oxidative stress (Bea et al., 2003).

## Ethylene And Interaction With H_2_O_2_

Ethylene has long been regarded as a stress hormone (Morgan and Drew, 1997). It is not only involved in plant growth and development, but also involved in plant responses to biotic stress, such as pathogen attack; and abiotic stress, such as wounding, ozone, and salinity (Abeles et al., 1992; Wang et al., 2009). Ethylene regulates many different processes in plants and has shown response in defense mechanism as well (Ecker, 1995). In order to evaluate the defensive role of ethylene against various environmental conditions signal transduction pathways for ethylene has been studied with mutants.

The roles of ethylene have been established in damage control caused by virulent bacteria or fungal pathogens when it is being inoculated (Bent et al., 1992; Lund et al., 1998) but its importance against avirulent bacteria infected plants has yet to be proven (Bent et al., 1992). The most important signaling molecules in the ethylene pathway are *ETR1* and *EIN2* (Buer et al., 2006). Change in gene expression of the ethylene receptor (*ETR1*) results in reduced ethylene response (O’Malley et al., 2005).

Environmental stress affects many signaling pathways in plants which also includes an alternative pathway (AP). Despite slight evidence about H_2_O_2_ and ethylene playing roles in inducing AP, there is no clear picture of how these signaling molecules are inducing the AP under various environmental conditions. Results of Wang et al. (2010) showed the possibility of involvement of H_2_O_2_ and ethylene mediated induction of AP under salt stress as it shows activity in wild-type callus whereas no activity was observed with *ETR1-3* callus. In recent years, an increasing number of positive results on ethylene toward mutants in *Arabidopsis* have confirmed its role in signaling pathways (Guo and Ecker, 2004). In another study H_2_O_2_ accumulation in simultaneous production of ethylene has been observed in tobacco plant stressed with ozone (Schraudner et al., 1998). In plants, oxygen-deficient conditions shifts energy metabolism from aerobic to anaerobic, which in turn adversely affects nutrient and water uptake. Eventually, hypoxiasignalling triggers the production of both hydrogen peroxide (H_2_O_2_) and ethylene. H_2_O_2_ and ethylene interplay has an effect on *AtERF73/HRE1* and *ADH1* expression during the early stages of hypoxia signaling in *Arabidopsis*. Hypoxia signaling induces the ethylene biosynthesis enzymes such as *ACC synthase* (*ACS*) and *ACC oxidase* (*ACO*) (Peng et al., 2005) (**Figure 3B**). *Arabidopsis AtERF73/HRE1* is very similar to the rice *Sub1A* and *SNORKEL* genes, which belongs to the group VII ERF (ethylene responsive factor) subfamily. They play major roles in the submergence tolerance of lowland and deepwater rice (Hattori et al., 2009). According to Yang (2014), *AtERF73/HRE1* positively regulates *ADH1* genes as well as negatively regulates *peroxidase* and *cytochrome* P450 genes in hypoxia signaling (**Figure 3B**).

## Conclusion

Increasing urge to identify the role of hydrogen peroxide as a signaling molecule has gathered the interest of researchers to focus their work on the mechanisms regulating the generation of hydrogen peroxide, and this is certainly an important growing area of research. Significant scientific effort in the last 10 years has determined the position of H_2_O_2_ in signal transduction networks in plants, demonstrating that it is essential for both the communication between external biotic and abiotic stimuli, and the control of developmentally regulated processes. There are many signaling pathways for H_2_O_2_ mediated stress and defense responses that have been studied, but it remains a large scope of additional research unexplored, which can further clarify the mechanism involved in these pathways. The focus should be imposed on a clear description of roles of endogenous compounds which modify the plant responses. It has been reported that the phytohormones like ABA, SA, JA, GA, and ethylene regulates the protective responses in plants under abiotic stress by involving in different H_2_O_2_ induced signaling. Despite of its regular activities in plant growth and development, ABA plays crucial role in H_2_O_2_ mediated stress cues. Zhang et al. (2007) indicated that ABA-induced H_2_O_2_ production mediates NO generation, which in turn, activates MAPK cascade and results in the over expression and up regulation in of antioxidant enzyme activities in ABA signaling. However, there are some contradictory roles of NO. According to Orozco-Cárdenas and Ryan (2002), NO has been shown to negatively modulate wound signaling in tomato plant blocking H_2_O_2_ production and proteinase inhibitor synthesis by JA, contradicting with previous study in which NO has been considered to show positive response in abiotic stress. Nevertheless, there are many studies suggesting H_2_O_2_ response in association with NO generation under biotic/abiotic stress (Delledonne et al., 2001; Romero-puertas and Sandalio, 2016).

Due to different results suggesting various roles of H_2_O_2_, it is important to focus future studies in getting a clear picture of signaling pathways during stress response in various conditions. Interactions between different signaling molecules and their biological functioning with the involvement in various pathways still needs to be cataloged. Another important aspect that should be focused on is the role and localization of enzymes, which are involved in signaling pathways. Some important factors for future research should be the identification of the site for H_2_O_2_ production in the cell and the major factors influencing its interaction with other signaling molecules.

## Author Contributions

ZC initiated the project. SS produced the figures. IS, SS, and ZC wrote the manuscript. SS and ZC revised the manuscript.

## Conflict of Interest Statement

The authors declare that the research was conducted in the absence of any commercial or financial relationships that could be construed as a potential conflict of interest.
